# Curing of Epoxy Resin DER-331 by Hexakis(4-acetamidophenoxy)cyclotriphosphazene and Properties of the Prepared Composition

**DOI:** 10.3390/polym11071191

**Published:** 2019-07-17

**Authors:** Evgeniy M. Chistyakov, Ivan V. Terekhov, Aleksey V. Shapagin, Sergey N. Filatov, Vladimir P. Chuev

**Affiliations:** 1 D. Mendeleev University of Chemical Technology of Russia, 125047 Moscow, Russia; 2 All-Russian Scientific Research Institute of Aviation Materials, 105005 Moscow, Russia; 3 A.N. Frumkin Institute of Physical Chemistry and Electrochemistry Russian Academy of Sciences, 119071 Moscow, Russia; 4 Belgorod National Research University, 308015 Belgorod, Russia

**Keywords:** phosphazenes, epoxy resin, noncombustibility, thermostability, amide cure, coke

## Abstract

The method of optical wedge revealed that the optimum temperature for compatibility of hexakis(4-acetamidophenoxy)cyclotriphosphazene (ACP) and DER-331 epoxy resin is in the range of 220–260 °C. The interdiffusion time of components at these temperatures is about 30 min. The TGA and differential scanning calorimetry (DSC) methods revealed the curing temperature of 280 °С for this composition. IR spectroscopy confirmed that the reaction between the resin and ACP is completed within 10 min. According to the DSC data, a glass transition temperature of 130 °С was estimated for the cured resin. Combustion test UL-94 demonstrated that the obtained material can be assigned to the fireproof category of V-0. Burning droplets were not formed during the burning. The coke formed during the combustion of samples possessed a dense and porous structure. The shape of pores is closed, while their size is in the range of 0.2–200 µm.

## 1. Introduction

The development of technologically simple and cost-efficient composite materials with unique properties, which can be applied in various fields of science and technology, is a hot topic in the modern scientific community [1,2,3].

Many such composites are based on epoxy resin due to its excellent mechanical properties, low dielectric permittivity, and good resistance to chemicals, solvents, and moisture [4]; but its high flammability hinders its practical applications [5]. Although traditional halogenated flame retardants have been designed to solve this problem, they have had to be replaced due to their health and environmental hazards by halogen-free analogs [6]. Organometallic compounds, especially organophosphorous ones [7,8,9], hold a prominent place in the production of such materials. For example, materials with reduced flammability were created from phosphorus-containing epoxy resin [10]. It was noted that with an increase in the content of the developed resin, the burning time of the samples decreased, and the yield of coke increased. A new superbranched phosphorus/nitrogen-containing flame retardant was investigated in another work [11]. The epoxy resin cured by this compound does not sustain combustion. The authors explain this by the fact that the structures of the coke of burned samples without the addition of flame retardant are fragmentary and broken. Coke of burned samples with modified resin has compact and continuous char layers. Another non-flammable material was obtained from anhydride-cured epoxy resin with the addition of 26.0 wt % of novel phosphorus/nitrogen containing polycarboxylic acid [12].

Among different phosphorus flame retardants, phosphazenes are of particular interest due to the synergistic effect of phosphorus and nitrogen atoms acting simultaneously [4,5,6,7,8,9,13,14,15,16,17,18,19,20,21,22]. Fireproof epoxy compositions based on phosphazenes were prepared, of which resistance to combustion was achieved by the addition of small amounts of phosphazene modifiers [4,5], e.g., hexa(4-maleimidophenoxy)cyclotriphosphazene (9 wt %) [13] or hexa-[4-(hydroxyanilinephosphaphenanthrenemethyl)-phenoxy]cyclotriphosphazene (10 wt %) [9]. Some phosphazene derivatives have proven themselves as hardeners of epoxy resins, for example, aminophosphazenes [16,17,22]. A novel compound containing active amine groups on polyphosphazene was successfully synthesized and applied as a reactive flame-retardant additive in epoxy resin [22]. It was synthesized from *N*-aminoethylpiperazine and hexachlorocyclotriphosphazene. A composite with 9.0 wt % of such phosphazene can pass the vertical burning tests and have a V-0 rating.

Amines are known as cured at low temperatures [23], which is not always convenient since the composition may be prematurely hardened during a long-term processing of the resin into a ware. At this end, amides are of considerable interest due to their less nucleophilic nitrogen atom, while the reaction between the amide and epoxy groups proceeds at higher temperatures [24].

Hexakis(4-acetamidophenoxy)cyclotriphosphazene (ACP) is the most actively explored among phosphazene derivatives, since its synthesis is fairly simple [25,26,27,28]. This compound may be of significant interest as a hardener of industrial epoxy resins for the production of non-combustible materials based on them.

## 2. Experimental Section

### 2.1. Materials and Methods

Hexachlorocyclophosphazene (HCP) (Fushimi Pharmaceutical Co., Ltd., Tokyo, Japan) was purified by the recrystallization from *n*-hexane with the consequent sublimation. The epoxy resin (DER-331) was purchased from DOW Chemical Company (Berlin, Germany), while the other reagents were obtained from Sigma-Aldrich (St. Louis, MO, USA). 4-Acetamidophenol and metallic sodium were used without any purification. Diglyme and THF were dried over CaH_2_ and distilled in vacuo. Ethanol was distilled over aluminum amalgam.

Thermal analysis of the obtained compounds was performed by synchronous thermogravimetric analysis (TGA) and differential scanning calorimetry (DSC) using a NETZSCH STA 449 F3 Jupiter instrument (Erich NETZSCH GmbH & Co. Holding KG, Selb, Germany) coupled with FT-IR Bruker Tensor 27 (Billerica, MA, USA) (10 °С min^−1^). Argon was used as a purge gas (70 mL/min). IR spectra were recorded on a Nicolet 380 FTIR spectrometer (Thermo Fisher Scientific, Waltham, MA, USA) equipped with the FTIR prefix spectrometer in the transmission mode in the range of 4000–400 cm^−1^. ^1^H, ^13^C, and ^31^P NMR spectra were recorded on a Bruker CXP–300 spectrometer (Billerica, MA, USA). To determine the size of ACP particles, a MSP-1 stereoscopic pancratic microscope equipped with a LOMO MS-5 digital camera and MCView software (AO Lomo, St. Petersburg, Russia) were used. Shear strength was measured on a PUMA-2 tensile testing machine (Tochmashpribor, Ivanovo, Russia). X-ray fluorescence analysis was performed on an ARL PFX-101 spectrometer (Thermo Fisher Scientific, Waltham, MA, USA). Elemental analysis was performed on an ЕА 1110 elemental analyzer (Antwerpen, Belgium).

### 2.2. Synthesis of Hexakis(4-acetamidophenoxy)cyclotriphosphazene (ACP)

ACP was synthesized according to a known procedure [28].

^1^H NMR (DMSO-*d*_6_, TMS, ppm): 9.90 (1H, –NH), 6.81–7.47 (4H, dd, Ar–H), 2.06 (3H, –CH_3_). ^13^C NMR (DMSO-*d*_6_, TMS, ppm): 168.2 (C=O), 145.1 (C–O), 136.6 (C–N), 120.7 (CH), 119.8 (CH), 24.0 (CH_3_). ^31^P NMR (DMSO-d_6_, ppm): 9.82 (s).

### 2.3. Determination of Compatibility and Interdiffusion for ACP and DER-331

The optical interferometry method was used to estimate the compatibility of ACP and DER-331. The measurements were carried out using an ODA-2 laser diffusiometer (Moscow, Russia) [29]. This method is based on the phenomenon of multibeam interference from two polished glass plate surfaces forming an angle of ≈2° between them. Inner surfaces of the glasses are covered with a layer of translucent metal possessing a high index of reflection.

ACP powder was placed between the glass plates and thermostated above its melting temperature, while DER-331 was injected into a wedge at the temperature of experiment. The moment of contact of the fronts was considered as the beginning of the diffusion mixing process.

The interdiffusion measurements performed in the isothermic mode. To estimate the compatibility of components, the temperature was raised and lowered in a stepped mode with a step of 10 °С in the range from 20 to 270 °С. The interdiffusion coefficients were calculated by the moving-boundary method at the laboratory coordinate system [30].

### 2.4. Preparation of the Composition Based on ACP and DER-331

The weight fraction of ACP relative to the resin was calculated according to the formula: *X* = *E* × *M*/(43 × *n*), wherein *E* is the weight fraction of epoxy groups in the resin, *M* is the molecular weight of ACP, 43 corresponds to the molecular weight of one epoxy group, and *n* is the functionality of ACP. Putting numbers into this equation gave 20 × 1035/(43 × 6) = 80.2 (%) (based on the mass of DER-331). The mass fractions of components used for the composition preparation were 44 and 56% for ACP and DER-331, respectively.

To prepare the samples, APC was ground in a mortar (the size of particles did not exceed 200 µm), the epoxy resin was added, and the mixture was ground again. The resulting paste was placed into a mold, vacuumized, heated to 220 °C, and held for 30 min at this temperature. The temperature was then elevated to 280 °С and maintained for 10 min.

### 2.5. Combustion Test of the Samples

The resistance to combustion for the prepared compositions was determined according to the UL-94 test.

### 2.6. Estimation of Gel Fraction

A weighed sample of the cured epoxy resin was placed into a paper bag and tightly closed. The bag with the sample was weighed and placed in a Soxhlet apparatus, and the soluble fraction was extracted with hot ethanol. Ethanol was selected since the both epoxy resin and ACP are soluble in it. Once the extraction was finished, the paper bag was removed from the apparatus, dried in vacuo at 100 °C, and weighed. The amount of gel fraction was calculated as the mass difference of the samples before and after extraction.

### 2.7. Investigation of Microstructure of the Coke Covers

A sample of composition taken after the combustion test was evaluated. To prepare it for the microstructural studies, the surface of the coke covers was cut from the sample. The resulting fragment was attached to the holder by a conductive carbon tape, and edges of the sample were covered with a silver-based glue. In a Q150R ES vacuum system (Quorum Technologies, Lewes, UK), a gold layer of 20 nm was then applied onto the sample. Microstructural analysis of the samples was carried out on a TESCAN VEGA 3 XMU (Brno, Czech Republic) scanning electron microscope (SEM) in the secondary electron (SE) mode. Quantitative processing of the obtained data was performed using the ImageScope Color software (Systems for microscopy and analysis, Moscow, Russia) designed for image analysis.

### 2.8. Elemental Analysis

The content of the elements in the ACP, DER-331, and the cured composition was calculated theoretically. To determine the phosphorus content in coke, X-ray fluorescence analysis was used. The content of C, N and H was determined according to ISO 17247: 2013.

### 2.9. Shear Strength Determination

Plates from carbon steel of the St-3 trademark (MMK, Magnitogorsk, Russia) were used to determine the shear strength of the adhesive bond. Composition for bonding was prepared and cured in accordance with Section 2.4. The tests were carried out according to ISO 4587.

## 3. Results and Discussion

**Figure 1:**
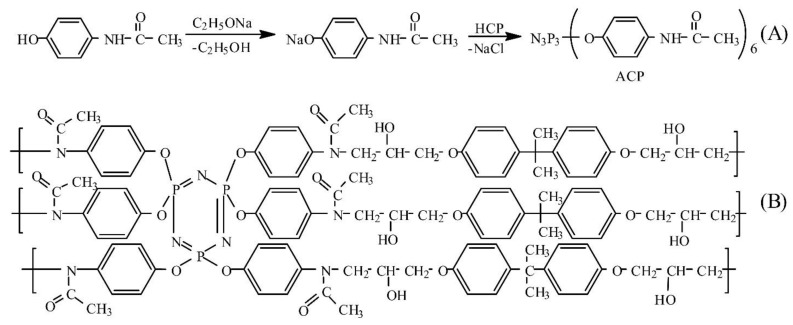
Synthesis of hexakis(4-acetamidophenoxy)cyclotriphosphazene (ACP) (**A**) and a proposed structure of the cured epoxy resin (**B**).

ACP was synthesized according to Scheme A (Figure 1). This compound can be an efficient curing agent for epoxy resins, since the ACP molecule contains six amide groups, and each of them can react with an oxirane cycle of the epoxy resin. This will result in a highly cross-linked polymer, whose structure may be represented by that shown in Figure 1B.

**Figure 2:**
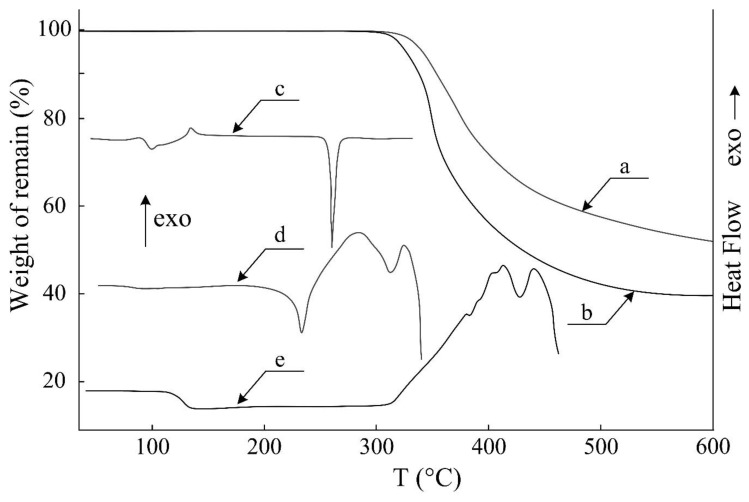
TGA curves for (**a**) cured composition and (**b**) mixture of ACP and DER-331; and differential scanning calorimetry (DSC) curves for (**c**) ACP, (**d**) mixture of ACP and DER-331, and (**e**) cured composition.

To evaluate the application of ACP as a hardener of epoxy resins, it was necessary to evaluate their mutual compatibility. Since ACP is a crystalline substance according to the DSC data (Figure 2c), solution-melts of ACP with DER-331 epoxy resin should be described by a constitutional diagram containing the crystalline equilibrium.

**Figure 3:**
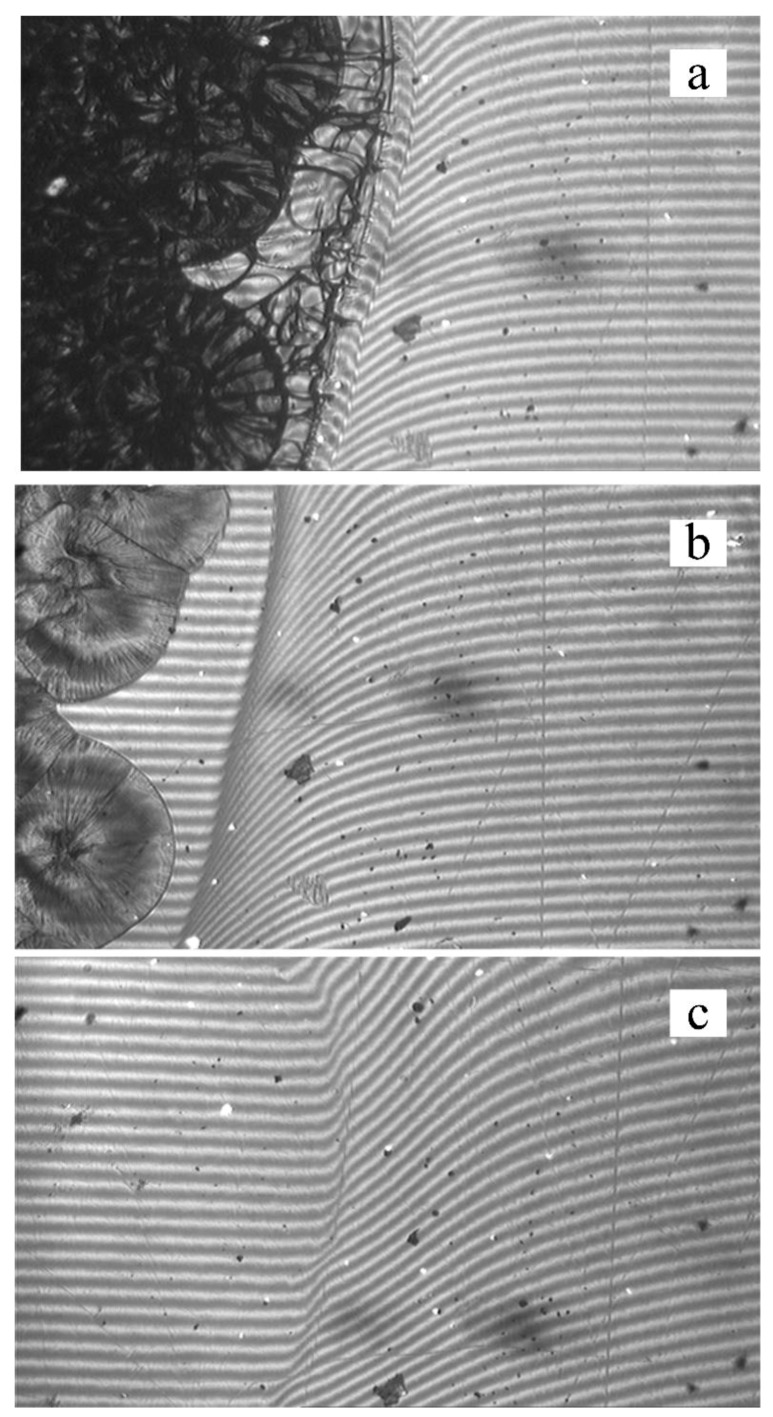
Interference patterns of the interdiffusion zones of the ACP–DER-331 system obtained at (**a**) 20, (**b**) 180 and (**c**) 270 °C.

Interdiffusion zones of the components were investigated by optical interferometry in a wide temperature range. Typical interference patterns at different temperatures are shown in Figure 3.

The complete compatibility of the components in the ACP–DER-331 system has been achieved above the melting point of ACP, which is evidenced by the resolved interference pattern characterized by a continuous concentration profile in the interdiffusion region (Figure 2c, and Video Abstract in Supplementary Materials). It should be noted that the crystallization of phosphazene was observed upon cooling the ACP solution in the resin down to 200 °C, which precludes obtaining a homogeneous system at the lower temperatures.

**Figure 4:**
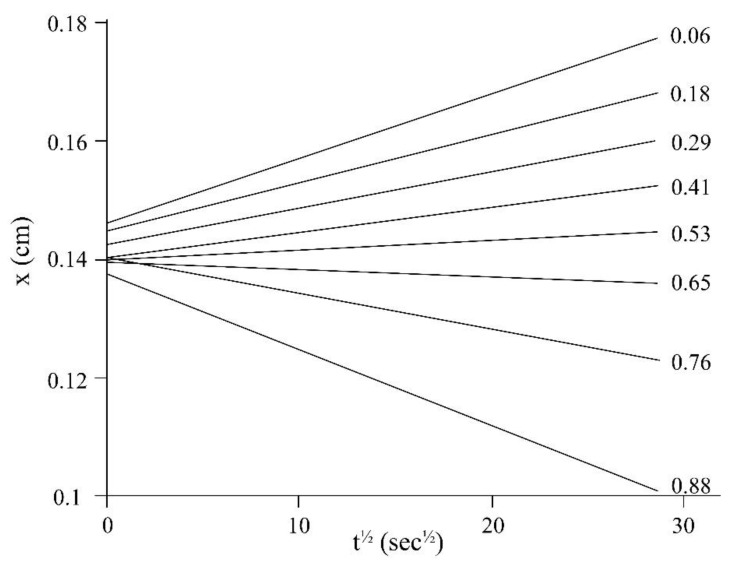
Typical kinetic dependences of the iso-concentration planes movement in the interdiffusion zone. The numbers indicate values of the mass fraction of DER-331.

Taking into account the movement of iso-concentration planes inside the interdiffusion zones (Figure 4), it was established that the process of mixing the components obeys the diffusion mechanism since it is described by the equation of *х*~*kt*^1/2^, wherein *k* is a constant associated with the interdiffusion coefficient.

**Figure 5:**
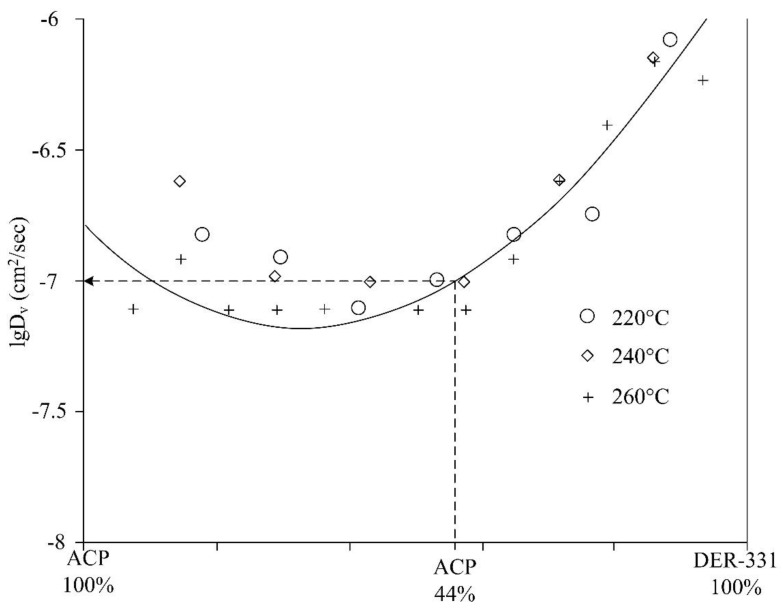
Concentration dependences of the interdiffusion coefficient on semi logarithmic scale at 220, 240, and 260 °С.

Taking into account the data shown in Figure 4, the concentration dependences of the diffusion coefficients were calculated at temperatures of 220, 240, and 260 °С. It was revealed that the dependences possess a similar character in the range of studied temperatures (Figure 5). It was shown that the values of the interdiffusion coefficients are 10^–6^ cm^2^ s^–1^ in the region of diluted ACP solutions and are nearly stable upon temperature changes. As the concentration shifts to the value of concentrated phosphazene solutions, the diffusion processes slows down to 10^–7^ cm^2^ s^–1^. It should be noted that in this region of concentration within the investigated temperature range, the magnitude of interdiffusion coefficients varies within the order of 0.5. Thus, in the area of limiting concentrations, the diffusion coefficient of ACP in DER-331 is one order of magnitude higher than that of DER-331 in ACP.

To prepare the composition based on ACP and DER-331, their fractions were calculated to be 44 and 56%, respectively. It should be noted that the diffusion rate at the ACP concentration of 44% in the epoxy resin does not change within the temperature interval shown in Figure 5, which can be explained by a depression of the ACP melting point. Melting of the ACP in the mixture begins at ~200 °C (Figure 2d), while the pure ACP melts only at 258 °С (see Figure 2c).

The duration of the dissolution process of ACP in DER-331 can be calculated from the calculated diffusion constants (Figure 5). Using the formula *t* = *R*^2^ × (2*D*)^−1^ (wherein *t* is the diffusion relaxation time, *D* is the diffusion coefficient, and R is the radius of ACP particle), one can conclude that ACP particles measuring 200 µm in size will dissolve in the resin at 220 °C in about 30 min.

**Figure 6:**
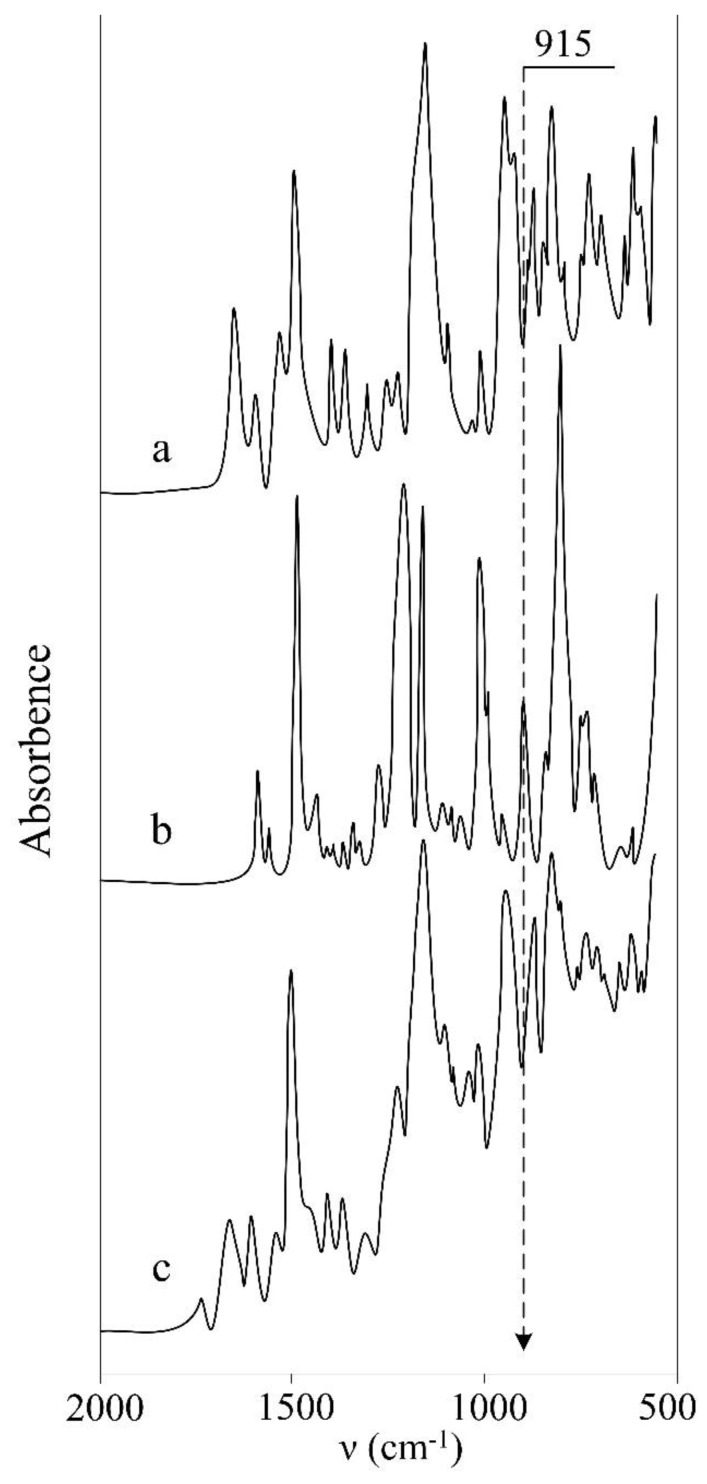
IR spectra of (**a**) ACP, (**b**) DER-331, and (**c**) cured composition.

The curing temperature of ACP–DER-331 composition was estimated by the DSC method. Figure 2d shows an exothermic effect above the melting point of ACP, which is caused by the beginning of the interaction between the components. The maximum of peak is at 280 °С, while a degradation of the material is already observable at 300 °C, which is also confirmed by TGA data (Figure 2b). At this end, the composition was cured at 280 °C for 10 min. In this interval of time, the interaction between the components was completed, which was confirmed by IR spectroscopy. The IR spectrum of epoxy resin (Figure 6b) contains a band of about 915 cm^–1^ corresponding to the asymmetric stretching vibrations of the ring, while this band is absent in the spectrum of ACP (Figure 6a) and disappears in the spectrum of cured composition (Figure 6c). The occurrence of reaction between the components was also confirmed by a high content (98%) of the gel fraction.

The shape of DSC curve for the cured resin (Figure 2e) is another confirmation that the reaction between components of the composition has completely proceeded under the reported conditions. This curve does not contain any thermal effects in the interval between the glass transition temperature of composition (130 °C) and the temperature corresponding to the beginning of its decomposition (300 °C, TGA, Figure 2b). It is known that the decomposition temperature of epoxy resin cured with aromatic amines depends little on the amount and type of flame retardant [11,22]. For DER-331 or E-44 epoxy resins, it is 300 °C. Therefore, the thermal stability of the cured ACP resin is quite satisfactory.

Results of the combustion test allowed us to assign the ACP-cured epoxy resin to the highest fireproof category V-0. The combustion time of vertically fixed samples upon the first and second exposures to flame was only 1 s, while burning droplets were not produced. It can be concluded that the resulting material is extremely non-flammable and exceeds phosphorus-nitrogen [11] and other phosphazene-containing flame retardants [16] in its flame-retardant properties.

**Figure 7:**
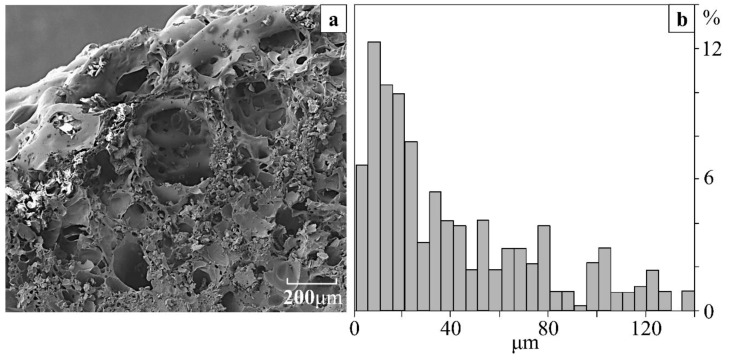
(**a**) SEM image of the coke cap and (**b**) the relative size distribution of pores after the combustion test of composition.

The coke covers formed after combustion of the cured resin was porous according to the data of microstructural studies and quantitative processing of the microphotographs (Figure 7a). The pores were closed, while their size varied in the range from 0.2 to 200 μm, wherein the smaller pores were predominant (Figure 7b).

**Figure 8:**
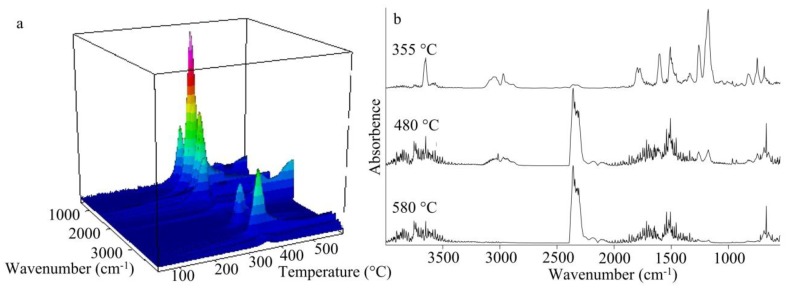
3D FTIR spectra of the pyrolysis gas products of ACP cured composition from TGA (**a**) and FTIR spectra of pyrolysis gas products at different temperatures (**b**).

The formed coke was dense, and its content at 600 °С was about 55% according to the TGA data (see Figure 2a). The TGA-FTIR spectra further disclosed the pyrolysis information (Figure 8). From Figure 8a it can be observed that the release rate of gas firstly has a big peak at 355 °C, then reduces slightly, and then has two more peaks at 480 and 580 °C. The curve at 355 °C belongs to the first degradation stage. The peak at 3622 cm^−1^ is the absorption of free hydroxyl group, and it indicates production of water and –OH containing organic compounds during the initial degradation. The peaks at 3040, 1600, 1190 and 760 cm^−1^ are typical absorptions of aromatic rings and phenol groups. The peaks at 2962 and 1350 cm^−1^ can indicate the presence of alkane groups. Both these groups of peaks confirm the presence of aromatic rings and aliphatic moieties, which can be attributed to isopropylphenol, formed from the DER-331 part of the polymer. The peaks at 2350 and 1790 cm^−1^ are typical for CO_2_, which started to form at this temperature. The peaks near 1350 and 1250 cm^−1^ can indicate the presence of C–N groups in the pyrolysis gas products. When the temperature rises up to 480 °C, the peaks of isopropylphenol can still be observed, but they become smaller. At the same time the peaks for water and CO_2_ are getting larger. At 580 °С the peaks show mainly water and CO_2_ in the pyrolysis gas products. A small peak at 1219 cm^−1^ at 580 °C can be considered as absorption of PO free radicals. From the elemental analysis of coke, it can be concluded (Table 1) that organic fragments undergo considerable destruction during the pyrolysis of the cured resin, as there is a significant reduction in the carbon and hydrogen content after pyrolysis. At the same time, the relative content of phosphorus atoms increases, while nitrogen content slightly decreases. As a result, it can be considered that the fragments at gas phase mainly come from the degraded organic parts of the polymer, and that phosphazene moieties of obtained polymer make a major contribution to thermal stability and coke formation. Thus, thermal stability can be controlled using different epoxy resins.

The developed composition on the basis of ACP has been tested as an adhesive for steel. The shear strength was 4 ± 0.1 MPa. At the same time, a cohesive break was observed. Cohesive break and a low value of tensile strength can be explained by the high brittleness of the cured resin. This disadvantage can be eliminated either through the introduction of fillers, or through the use of other types of resins, which is the subject of further research.

## 4. Conclusions

In summary, the composition based on the industrial epoxy resin DER-331 cured with ACP (a phosphazene containing amide groups) has been designed and prepared. The obtained resin possesses the glass transition temperature of 130 °C, the decomposition temperature of 300 °C and the shear strength of 4 MPa. Moreover, this composition demonstrated very high flame-retardant properties. The damping time after the first and second exposures to flame was only 1 s, which is probably due to the porous and dense structure of the formed coke, as well as its high content. Since the coke is incombustible, it creates a protective layer and contributes to the damping of the resin. It also hinders heat propagation in the sample, thereby preventing the thermal decomposition of unaffected areas of the material and the release of low molecular weight combustible substances that are the main source of flames. The pores inside the formed coke improve the thermal insulation of the material; therefore, an increase in the number of closed pores prevents the spread of flames. All this makes the developed composition promising for practical use as non-flammable thermally stable adhesives and matrices for different compositions.

## Figures and Tables

**Table 1:** The elemental content of the substances (wt. %).

Element	ACP	DER-331	Cured Resin	Сoke
C	55.65	72.12	64.82	42.09
O	18.55	18.82	18.68	–
H	4.64	7.06	5.84	2.00
N	12.16	–	6.14	5.17
P	9.00	–	4.54	9.40

## Supplementary Materials

The following are available online at https://www.mdpi.com/2073-4360/11/7/1191/s1, The raw/processed data required is presented in the manuscript and Video Abstract.
