# Supplementary figures and images for: Curing of Epoxy Resin DER-331 by Hexakis(4-acetamidophenoxy)cyclotriphosphazene and Properties of the Prepared Composition
Source: Polymers (Basel). 2019 Jul 17;11(7):1191. doi: 10.3390/polym11071191 (PMC6680891; doi:10.3390/polym11071191)

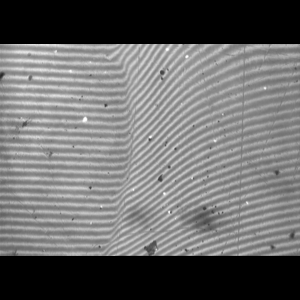

Supplement: Supplementary file 1 [file polymers-11-01191-s001.zip › polymers-528098-SI.gif]
